# Bone marrow-derived mesenchymal stromal cells obstruct AML-targeting CD8^+^ clonal effector and CAR T-cell function while promoting a senescence-associated phenotype

**DOI:** 10.1007/s00262-023-03594-1

**Published:** 2024-01-17

**Authors:** Russell Towers, Lidia Trombello, Maximilian Fusenig, Antje Tunger, Anna-Lena Baumann, Roberto Savoldelli, Rebekka Wehner, Frederick Fasslrinner, Claudia Arndt, Francesco Dazzi, Malte Von Bonin, Anja Feldmann, Michael P. Bachmann, Manja Wobus, Marc Schmitz, Martin Bornhäuser

**Affiliations:** 1 Medical Clinic 1 (MK1), University Hospital Carl Gustav Carus, TU Dresden, Fetscherstraße 74, 01307 Dresden, Germany; 2 National Centre for Tumor Disease (NCT/UCC), Fetscherstraße 74, 01307 Dresden, Germany; 3 Sant’Anna School of Advanced Studies, Piazza Martiri della Libertà 33, 56127 Pisa, Italy; 4 University of Pisa, Lungarno Antonio Pacinotti 43, 56126 Pisa, Italy; 5 Max Bergmann Center of Biomaterials Dresden, Leibniz Institute of Polymer Research Dresden e.V., Hohe Straße 6, 01069 Dresden, Germany; 6 Faculty of Medicine Carl Gustav Carus, Institute of Immunology, TU Dresden, Fetscherstraße 74, 01307 Dresden, Germany; 7 School of Cancer and Pharmaceutical Research, Kings College, London, SE5 9RS UK; 8 Partner Site Dresden, and German Cancer Research Center (DKFZ), German Cancer Consortium (DKTK), Im Neuenheimer Feld 280, 69120 Heidelberg, Germany; 9 Department of Radioimmunology, Helmholtz Center Dresden-Rossendorf, Institute of Radiopharmaceutical Cancer Research, Bautzener Straße 400, 01328 Dresden, Germany; 10 Faculty of Medicine Carl Gustav Carus, Mildred Scheel Early Career Center, TU Dresden, Fetscherstraße 74, 01307 Dresden, Germany

**Keywords:** Tumor microenvironment, Immunomodulation, Immunotherapy, T-lymphocytes

## Abstract

Bone marrow mesenchymal stromal cells (MSCs) have been described as potent regulators of T-cell function, though whether they could impede the effectiveness of immunotherapy against acute myeloid leukemia (AML) is still under investigation. We examine whether they could interfere with the activity of leukemia-specific clonal cytotoxic T-lymphocytes (CTLs) and chimeric antigen receptor (CAR) T cells, as well as whether the immunomodulatory properties of MSCs could be associated with the induction of T-cell senescence. Co-cultures of leukemia-associated Wilm’s tumor protein 1 (WT1) and tyrosine-protein kinase transmembrane receptor 1 (ROR1)-reactive CTLs and of CD123-redirected switchable CAR T cells were prepared in the presence of MSCs and assessed for cytotoxic potential, cytokine secretion, and expansion. T-cell senescence within functional memory sub-compartments was investigated for the senescence-associated phenotype CD28^−^CD57^+^ using unmodified peripheral blood mononuclear cells. We describe inhibition of expansion of AML-redirected switchable CAR T cells by MSCs via indoleamine 2,3-dioxygenase 1 (IDO-1) activity, as well as reduction of interferon gamma (IFNγ) and interleukin-2 (IL-2) release. In addition, MSCs interfered with the secretory potential of leukemia-associated WT1- and ROR1-targeting CTL clones, inhibiting the release of IFNγ, tumor necrosis factor alpha, and IL-2. Abrogated T cells were shown to retain their cytolytic activity. Moreover, we demonstrate induction of a CD28^lo^CD27^lo^CD57^+^KLRG1^+^ senescent T-cell phenotype by MSCs. In summary, we show that MSCs are potent modulators of anti-leukemic T cells, and targeting their modes of action would likely be beneficial in a combinatorial approach with AML-directed immunotherapy.

**Supplementary Information:**

The online version contains supplementary material available at 10.1007/s00262-023-03594-1.

## Introduction

Acute myeloid leukemia (AML) is a form of hematological cancer derived from the abnormal expansion of myeloid precursor cells, resulting in invasion of the bone marrow and ultimately failure of normal hematopoiesis. As it stands, the 5-year survival rate is less than 30%, underlining a desperate need for alternative treatments. Chimeric antigen receptor (CAR) T cells are a promising avenue currently in early clinical trials [1]. Initial reports have noted however that while several refractory/relapsed AML patients achieve a response to CAR T-cell adoption, only a minority achieve complete remission and a relevant proportion fail to respond at all, highlighting the need for further research into mechanisms of resistance [2–4]. In addition to immunosuppressive pathways intrinsic to AML blasts, neighboring constituents of the bone marrow can provide additional protection. In particular, a lot of interest has been garnered for resident mesenchymal stromal cells (MSCs) which were found to possess an impressive immunomodulatory capacity in suppressing the proliferation and the inflammatory potential of T cells [5]. This along with other properties such as multipotency has highlighted the therapeutic potential of MSCs in regenerative medicine and the treatment of immune disorders [6].

It follows to ask if MSCs can interfere with the action of T-cell-based immunotherapies in the context of hematological malignancy. At the time of writing, few reports have come out addressing this topic. One early report investigated whether MSCs could ablate the cytotoxic potential of T cells specific for the AML antigen Wilm’s tumor protein 1 (WT1), of which they found no effect [7]. More recently, it was reported that infusion of MSCs did not affect CD19 CAR T-cell activity in acute lymphocytic leukemia xenografts despite being able to efficiently control inflammation in a acute colitis murine model [8]. In contrast, another study demonstrated that MSCs could in fact decrease cell lysis mediated by several CAR T-cell constructs against multiple myeloma [9]. To reconcile these contradictory reports, it is apparent that more research in the field is needed.

To gain deeper insight into this issue, firstly, we investigated the effect of MSCs on the proliferative, secretory, and cytotoxic potential of clonal CD8^+^ cytotoxic T-lymphocytes (CTLs) specific for the leukemia-associated antigens WT1 and tyrosine-protein kinase transmembrane receptor 1 (ROR1) [10, 11], as well a switchable CAR T-cell system redirected against the AML marker CD123 currently tested in a phase I clinical trial [12–15]. We demonstrate that MSCs induce a reduction in T-cell proliferation and release of inflammatory cytokines, but without ultimately influencing killing potential against AML targets. Secondly, aberrant senescent T cells have been identified in circulation and within the bone marrow of AML patients [16–18], and we demonstrate here that MSCs can induce the senescence-associated CD28^lo^CD27^lo^CD57^+^KLRG1^+^ phenotype in T cells.

## Methods

### Isolation of mesenchymal stromal cells and peripheral blood mononuclear cells

MSCs were isolated from bone marrow aspirates of healthy donors (EK307082018) and AML patients (EK98032010) after informed consent and approval by the local review board, as previously described [19]. MSCs were characterized based on the criteria set out by the International Society for Cellular Therapy [20]. Peripheral blood mononuclear cells (PBMCs) were isolated by density gradient centrifugation using Pancoll (PAN-Biotech, Germany) from blood donated by healthy volunteers (EK206082008).

### PBMC co-culture

PBMCs and MSCs were co-cultured at a 5:1 or 100:1 ratio with CD3/CD28 Dynabeads (Gibco, USA) in RPMI: Roswell Park Memorial Institute 1640 (Gibco, USA) with 10% fetal bovine serum (FBS) (Merck, Germany). Proliferation assays were carried out as previously described [19]. The Transwell Permeable Support system with 0.4-μm polycarbonate membranes (Corning, USA) was used for non-contact cultures.

### CTL clone co-culture

CTL clones were generated as described elsewhere [10, 11]. Matched HLA-A*02:01 T2 target cells were pulsed with 20 μg/mL WT1_126_ (RMFPNAPYL) or HIV Gag-Pol_896_ (ILKEPVHGL) nonamers and HLA-B*07:02 K562 with ROR1_783_ (NPRYPNYMF) or HIV Gag-Pol_355_ (GPGHKARVL) nonamers. For cytotoxicity assays, 100 μCi/mL ^51^Chromium (Hartmann Analytic, Germany) was added. CTLs and target cells were co-cultured with MSCs at an E:T:MSC ratio of 10:1:2 in RPMI. Supernatant was collected and mixed with Ultima Gold scintillation cocktail (PerkinElmer, USA), and ^51^Cr release was assessed with the MicroBeta 2 (PerkinElmer, USA). Supernatant was also collected for cytokine determination.

### Switchable CAR T-cell co-culture

The switchable CAR T-cell system has been described elsewhere [12]. AML blasts were collected from a primary patient sample derived from a 74-year-old female diagnosed with NPM-1 and CEBPA-mutated AML. Phenotyping revealed > 90% positivity for CD123 within the marrow blast population. The patient had provided written consent to the banking of the material and to its use within the research project (EK98032010). Target MOLM-13 and AML blasts were labeled with eFluor 670 (Thermo Fisher, USA) and co-cultured with CAR T cells in the Transwell Permeable Support system with MSCs at a E:T:MSC ratio of 16:16:3 with 0.5-nM target module (TM) in RPMI/Complete (RPMI with 10% FBS, 100 μg/ml penicillin/streptomycin, 1% nonessential amino acids, 2-mM N-acetyl-l-alanyl-l-glutamine, and 1-mM sodium pyruvate (Biochrom, UK)). For indoleamine 2,3-dioxygenase 1 (IDO-1) blocking assays, 0.2-mM 1-methyl-L-tryptophan (1-MT) (Sigma-Aldrich, USA) diluted in 0.2-mM NaOH was added. For cytotoxicity determination, CAR T cells (PI^−^eGFP^+^) and target cells (PI^−^eFluor670^+^) were quantified with the MACSQuant X (Miltenyi Biotec, Germany). MSC viability was assessed with the PE Annexin V Apoptosis Detection Kit (BD, USA). Supernatant was collected for cytokine analysis. Fresh RPMI/Complete with 0.5-nM TM was added back to the cultures after each collection. Cultures were restimulated twice with target cells on day 2 and day 4 or 5.

### Cytokine assessment

Cytokine concentrations were assessed with commercial kits. Human IFNγ ELISA Set and Human IL-2 ELISA Set (BD, USA) were measured on the Sunrise spectrophotometer and analyzed with Magellan software (Tecan, Switzerland). Interleukin (IL)-2, IL-17a, tumor necrosis factor alpha (TNFα), interferon gamma (IFNγ), soluble Fas ligand (sFasL), granzyme A, and perforin were measured with the LEGENDplex Human CD8/NK Panel (Biolegend, USA) on the MACSQuant X (Miltenyi Biotec, Germany) and analyzed with Legendplex software (Biolegend, USA).

### Gene and cell surface protein expression

For cell surface protein expression, MSCs, PBMCs, and CAR T cells were resuspended with fluorochrome-labeled antibodies (Supplementary Table 1) and 40 ng/mL DAPI, acquired on the LSR II flow cytometers (BD, USA), and analyzed with FlowJo software (BD, USA).

For gene expression, RNA was isolated from MSCs with TRIzol reagent (Thermo Fisher, USA), and cDNA libraries were prepared with RevertAid First Strand cDNA Synthesis Kit (Thermo Scientific, USA). Quantitative polymerase chain reaction (qPCR) was performed with the Maxima SYBR Green/ROX qPCR Master Mix (Thermo Scientific, USA) on the QuantStudio 3 (Applied Biosystems, USA). Primers are listed in Supplementary Table 2.

### Statistical analyses

Standard deviation (SD) and one-way or two-way ANOVA with Bonferroni’s correction for multiple comparisons were calculated with Graphpad Prism 6. *p* values ≤ 0.05 were considered statistically significant and were further stratified: *p* ≤ 0.05 (*), *p* ≤ 0.01 (**), *p* ≤ 0.001 (***), and *p* ≤ 0.0001 (****).

## Results

### MSC-mediated interference of anti-leukemic CD8^+^ T-cell activity

**Fig. 1:**
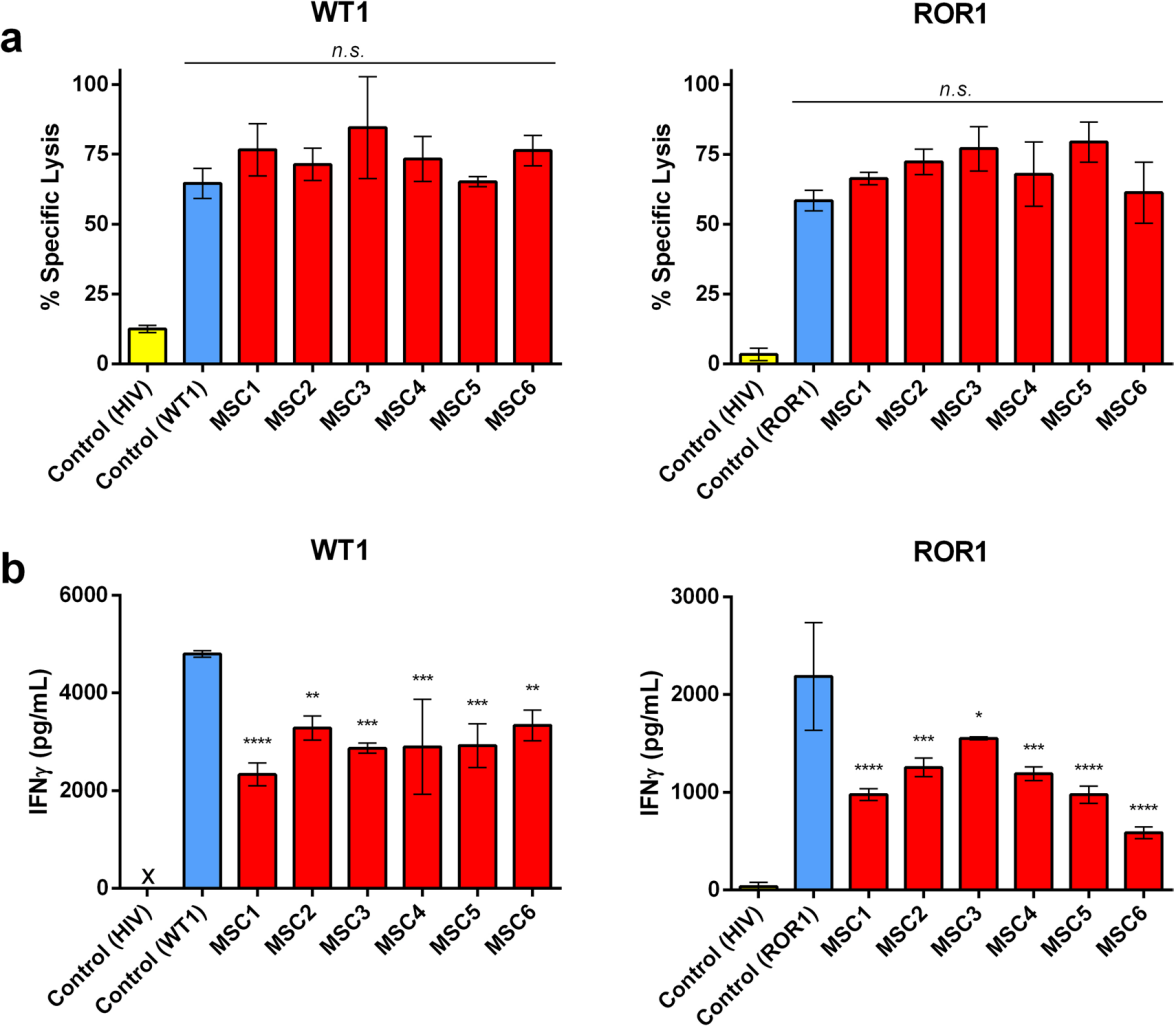
MSCs modulate the inflammatory capabilities of WT1- and ROR1-reactive CD8+ T-cell clones without affecting cytotoxicity. **a** Antigen-specific WT1- and ROR1-reactive CTL clones were, respectively, incubated with 51Cr-loaded target T2 cells pulsed with WT1 peptide or K562 cells pulsed with ROR1 peptide in the presence or absence of 1 × 104 allogeneic MSCs from six healthy donors (MSC1-6) at a E:T:MSC ratio of 10:1:2. Target cells pulsed with irrelevant HIV Gag-Pol peptides served as a negative control. Efficacy of CTL-mediated lysis of target cells was determined by 51Cr release after 4 h as measured by *β*-counter and is expressed as a percentage of maximum lysis with correction for spontaneous release. **b** Cell cultures were prepared as in (**a**) without 51Cr loading. After 24 h, supernatants were collected and assessed for IFNγ concentration via ELISA. Data are representative of three independent experiments and are presented as the mean of technical triplicates ± SD. Asterisks represent statistically significant differences compared to the control (* *p* ≤ 0.05; ** *p* ≤ 0.01; *** *p* ≤ 0.001; and **** *p* ≤ 0.0001; n.s. not significant)

MSCs from healthy donors were selected for their ability to inhibit PBMC proliferation in co-culture (Supplementary Fig. 1). Next, we evaluated the immunomodulatory potential of these MSCs on the cytotoxic capabilities of WT1- and ROR1-specific CD8^+^ CTL clones, and Fig. 1a shows that the MSCs did not significantly affect cytotoxicity of the CTLs against their target cells. MSCs are also reported to decrease the release of inflammatory cytokines such as IFNγ, TNFα, and IL-2. Despite having no discernable effect on cytotoxicity, IFNγ release was significantly decreased after 24 h in the presence of MSCs for both CTL clones (Fig. 1b). The decrease in IFNγ secretion by ROR1-reactive CTLs could even be observed as early as 4 h of co-culture (Supplementary Fig. 2a), along with other cytokines (IL-2 and TNFα). In contrast, MSCs did not have a significant effect on the concentrations of the cytotoxic effector molecules sFasL, granzyme A, or perforin within these same cultures, corroborating the cytotoxicity data (Supplementary Fig. 2b).

### MSC-mediated interference of AML-retargeted switchable CAR T-cell activity

**Fig. 2:**
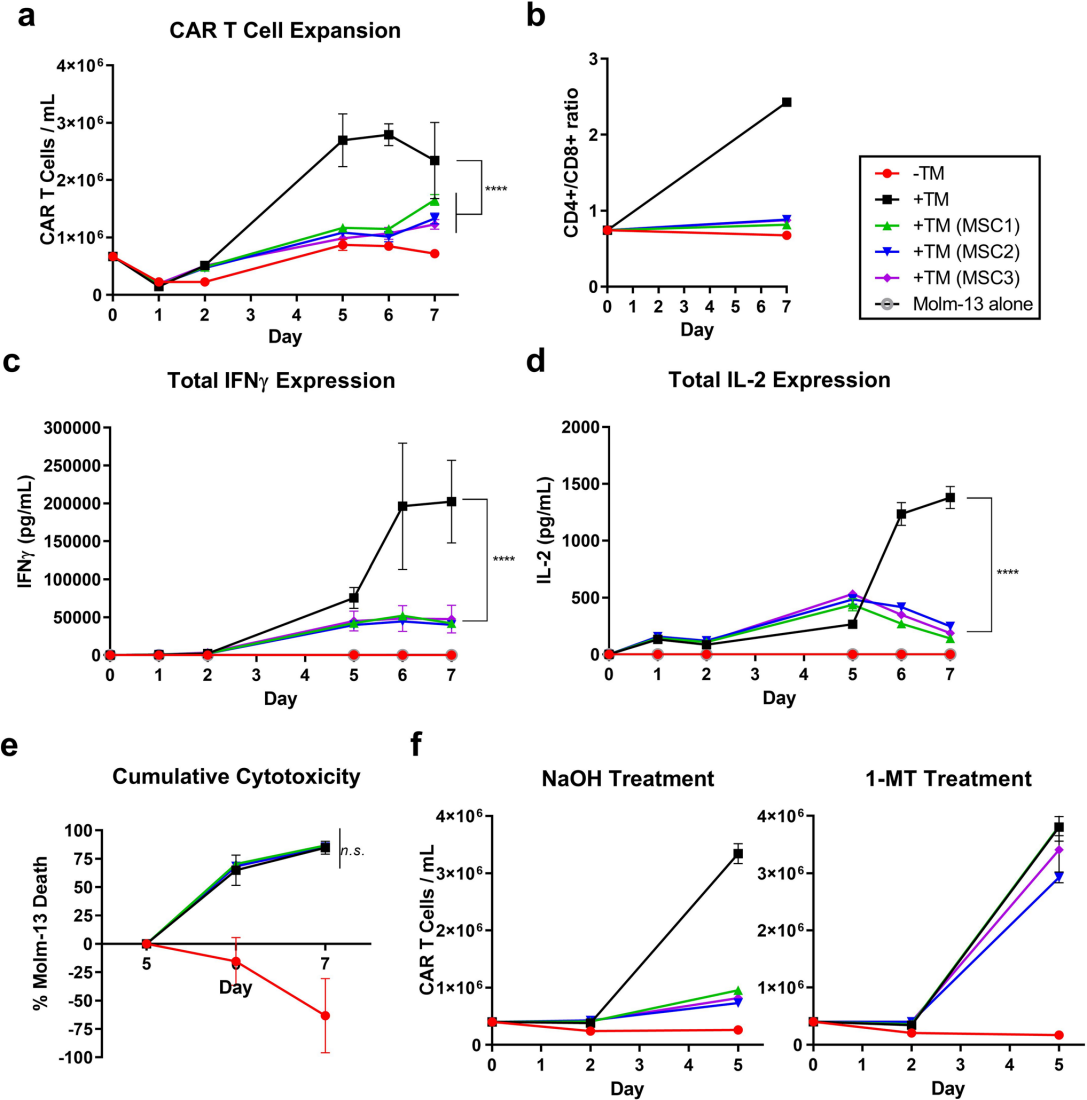
MSCs interfere with the proliferative and inflammatory capabilities of CD123-targeting switchable CAR T cells without affecting cytotoxicity. In the apical chamber of the Transwell cell culture system, eGFP^+^ CAR T cells were redirected with an anti-CD123 target module (+ TM, 0.5 nM) against eFluor670-labeled MOLM-13 cells in the presence or absence in the basolateral chamber of 7.5 × 10^3^ MSCs from three allogeneic healthy donors (MSC1-3) at a E:T:MSC ratio of 16:16:3. Cultures without TM (-TM) served as a negative control. Additional eFluor670-labeled MOLM-13 cells were added on day 2 (1.2 × 10^5^) and day 5 (variable at a E:T of 1:2). Surviving CAR T cells (PI^−^eGFP^+^) and MOLM-13 cells (PI^−^eFluor670^+^) were quantified via flow cytometry at 1, 2, 5, 6, and 7 days of culture. Shown are representative results of three independent assays. Data points are presented as the mean of technical triplicates ± SD. Asterisks represent statistically significant differences (**** *p* ≤ 0.0001; *n.s.* not significant). **a** CAR T-cell expansion was assessed as PI^−^eGFP^+^ cells/mL over time. **b** CD4^+^ and CD8^+^ CAR T-cell populations were assessed by flow cytometry on days 0 and 7. Data are presented as the ratio of CD4^+^/CD8^+^ fractions in the DAPI^−^eFluor670^−^CD45^+^eGFP^+^ cell population. **c** and **d** Supernatant was collected on days 1, 2, 5, 6, and 7 and assessed for IFNγ and IL-2 concentration via ELISA. **e** MOLM-13 killing kinetics after the third round of CAR T-cell stimulation on day 5 at a E:T of 1:2. Data points represent cumulative loss of target cells relative to the initial population on day 5. Negative values indicate MOLM-13 proliferation. **f** In a separate assay, co-cultures were treated with 0.2-mM NaOH (left) or 0.2-mM 1-MT/NaOH (right). CAR T-cell expansion was assessed as DAPI-eGFP + cells/mL over time

**Fig. 3:**
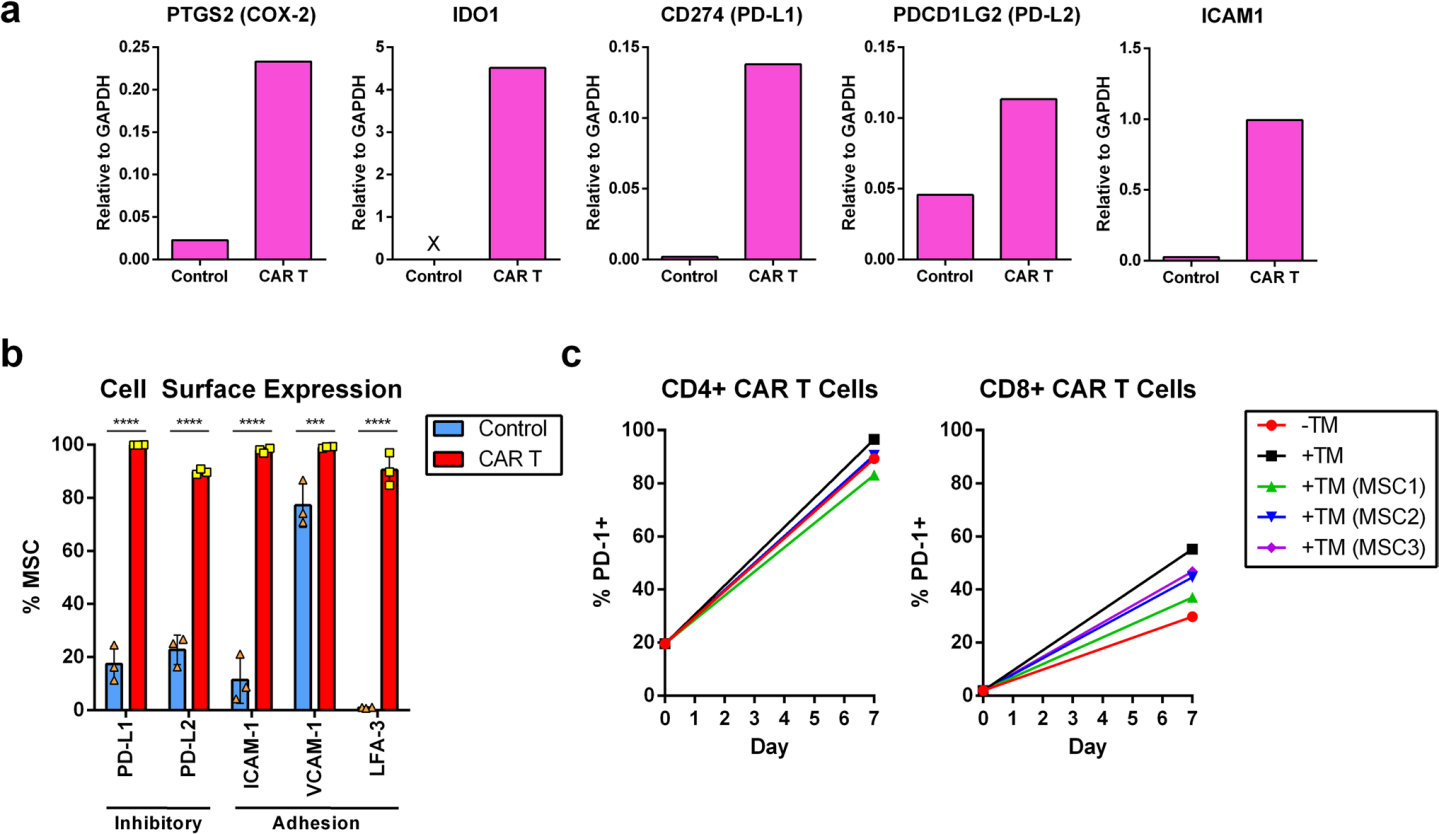
AML-activated CAR T-cell inflammatory stimuli induce the expression of immune checkpoint and lymphocyte adhesion molecules in MSCs. Co-cultures were prepared as in Fig. 2. **a** On day 7, mRNA was isolated from the MSCs from a single donor and reverse-transcribed into cDNA libraries. The expression of the indicated genes was assessed by qPCR, and the data are reported as expression levels relative to that of GAPDH. **b** and **c** On day 7, cells were collected and analyzed for cell surface expression of the indicated markers by flow cytometry. Shown are representative results of two independent experiments. b Cell surface expression of the indicated markers on DAPI^−^ MSCs of the basolateral chamber. Data are presented as the mean % of positive MSCs from biological triplicates ± SD. Asterisks represent statistically significant differences (*** *p* ≤ 0.001 and **** *p* ≤ 0.0001). c Cell surface expression on day 0 and day 7 of PD-1 on CD4^+^ (left) and CD8^+^ (right) CAR T cells of the apical chamber (DAPI^−^eGFP^+^CD45^+^). Data are presented as the mean % of PD-1 positive CAR T cells

The flow cytometry examination of the immunomodulatory potential of MSCs against switchable CAR T cells was conducted long term with repeated cytotoxic challenge in the Transwell cell culture system; MSCs and CAR T cells were thus limited to paracrine interactions. CAR T cells redirected against MOLM-13 cells with the anti-CD123 TM had significantly inhibited expansion kinetics in MSC co-cultures, approaching the minimal growth rate of the CAR T cells lacking TM-mediated stimulation (Fig. 2a). Intriguingly, the presence of MSCs abrogated the increased expansion of CD4^+^ CAR T cells compared to their CD8^+^ counterparts, maintaining a steady CD4^+^/CD8^+^ ratio over time similar to the unstimulated cultures (Fig. 2b). MSCs also decreased secretion of IFNγ and IL-2 by the CAR T cells, as observed previously with the CTL clones (Fig. 2c and d). Finally, the cytotoxic capabilities of the CAR T cells were studied by measuring the kinetics of MOLM-13 death after the third round of cytotoxic challenge. In line with the analysis of CTL clones, MSCs did not have an observable effect on the killing capacity of the CAR T cells, despite inducing overall lower proliferative and cytokine secretion capabilities (Fig. 2e). Similar trends could be observed when using patient-derived MSCs and AML blasts (Supplementary Fig. 3). In response to CAR T-cell activity, a large increase in gene expression was observed in MSCs for IDO-1 (Fig. 3a). To study the contribution of IDO-1 on suppression of CAR T-cell expansion, co-cultures were treated with the IDO-1 inhibitor 1-MT. In response, inhibition of expansion was almost completely reversed (Fig. 2f). To verify that this was not due to toxicity of the treatment, MSCs were subsequently assessed for apoptosis and necrosis and found to remain viable (Supplementary Fig. 4).

In addition to IDO-1, increase in gene expression in response to CAR T-cell activity was observed for the immunosuppressive factor cyclooxygenase 2 (COX-2), immune checkpoint ligands programmed cell death ligand 1 (PD-L1) and PD-L2, and intercellular adhesion molecule 1 (ICAM-1) (Fig. 3a). PD-L1, PD-L2, and ICAM-1, along with vascular cell adhesion protein 1 (VCAM-1) and lymphocyte function-associated antigen 3 (LFA-3), were further validated for cells surface expression (Fig. 3b). In parallel, programmed cell death 1 (PD-1) also increased significantly on the cell surface of CAR T cells over time (Fig. 3c).

### Induction of the T-cell senescence-associated phenotype by MSCs

**Fig. 4:**
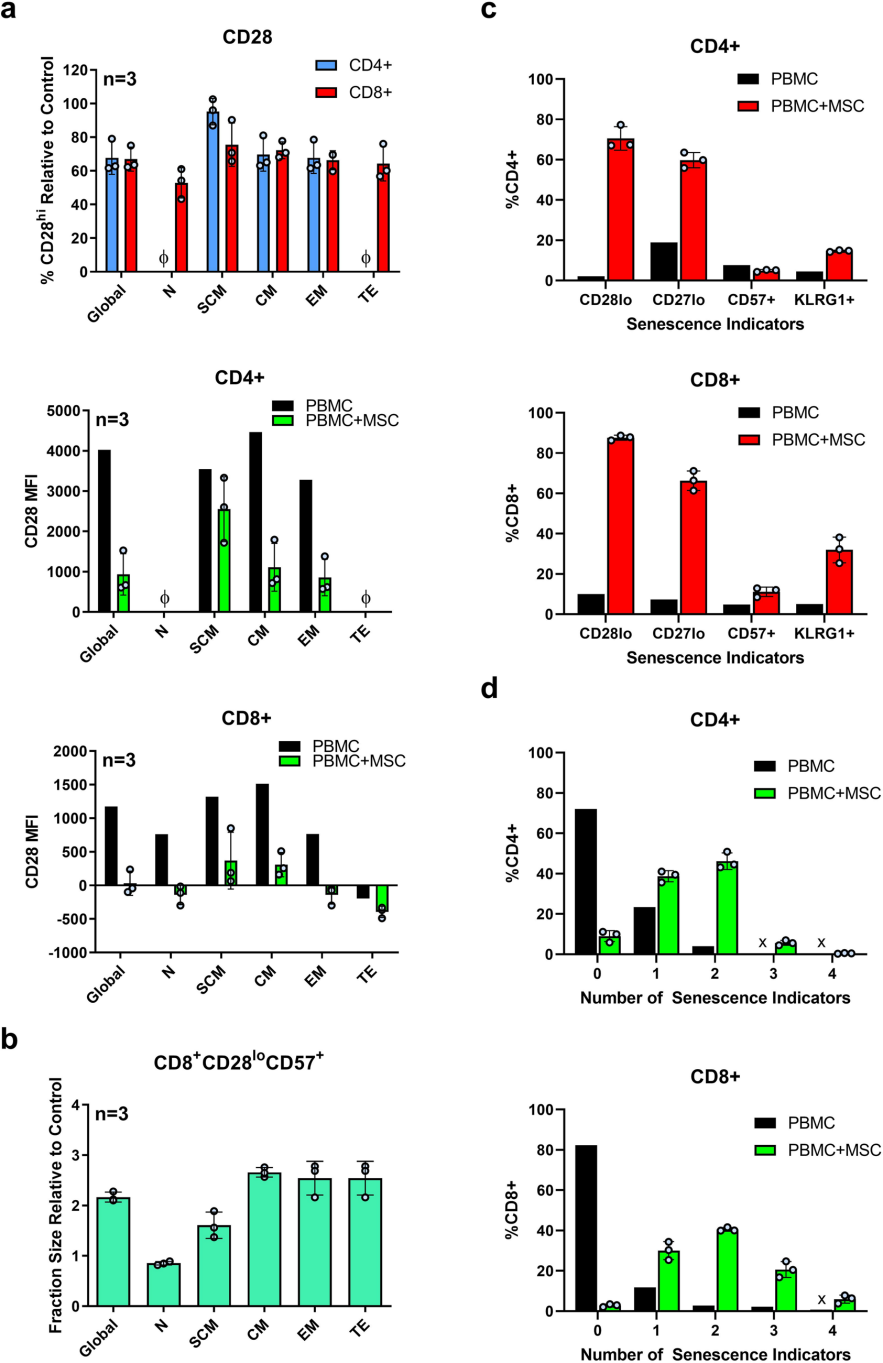
MSCs induce senescence of unmodified T cells, as characterized by loss of CD28 and CD27, and gain of CD57 and KLRG1. Healthy donor PBMCs were cultured with or without 5 × 10^4^ allogeneic MSCs from three healthy donors (MSC1-3) at a PBMC:MSC ratio of 5:1 and stimulated with anti-CD3/CD28 antibody-coated beads. After 6 days, cells were harvested and analyzed by flow cytometry. T-cell senescence (CD28^lo^, CD27^lo^, CD57^+^, KLRG1^+^) was assessed within the global CD4^+^ and CD8^+^ T-cell populations (global; DAPI^−^CD45^+^CD4^+^ and DAPI^−^CD45^+^CD8^+^), as well as within further T-cell memory subpopulations: naïve (N; CD45RA^+^CCR7^+^CD45RO^−^), stem cell memory (SCM; CD45RA^+^CCR7^+^CD45RO^+^), central memory (CM; CD45RA^−^CCR7^+^CD45RO^+^), effector memory (EM; CD45RA^−^CCR7^−^CD45RO^+^), and terminal effector (TE; CD45RA^+^CCR7^−^CD45RO^−^). **a** Above: Fraction size of CD28^hi^ cells within the global and memory stages of CD4^+^ (blue) and CD8^+^ (red) T-cell population in direct MSC co-cultures relative to the control. Below: Median fluorescence intensity (MFI) of CD28 within the global and memory stages of CD4^+^ (above) and CD8^+^ (below) T-cell populations for control (black) MSC co-cultures (green). Data are presented as the mean of biological triplicates ± SD. φ indicates lack of sufficient number of events for assessment. **b** Fraction size of CD28^lo^CD57^+^ cells within the global and memory stages of the CD8^+^ T-cell population in direct MSC co-cultures relative to the control. Results are representative of three independent experiments. **c** Fraction size of each senescence marker within the global CD4^+^ (above) and CD8^+^ (below) populations for control (black) and MSC co-cultures (red). **d** Fraction size based on the number of co-expressed senescence indicators within the global CD4^+^ (above) and CD8^+^ (below) populations for control (black) and MSC co-cultures (green). Data are presented as the mean of biological triplicates ± SD

In addition to PD-1, investigation of co-receptor CD28 expression on the surface of activated CAR T cells was also conducted, revealing MSC-mediated decrease in CD28 median fluorescence intensity (MFI) (Supplementary Fig. 5). Using PBMCs, we investigated whether MSCs could induce an enrichment of senescence-associated CD28^−^ and CD57^+^ T cells within the global CD4^+^ and CD8^+^ populations, as well as within further sub-compartments of T-cell differentiation and memory as defined by cell surface expression of CD45RA, CCR7, and CD45RO. Much like with the CAR T cells, MSC-mediated loss of CD28 could be observed on T cells within both the CD4^+^ and CD8^+^ fractions, as well as memory subfractions though CD4^+^ naïve and CD8^+^ terminal effector T cells remained largely unaffected (Fig. 4a). When referencing senescent T cells as CD8^+^CD28^−^CD57^+^ [16], we measured average fold changes ranging from 0.9 to 3.7 compared to the control within the differentiation subpopulations (Fig. 4b). Supplementary Fig. 6 shows example contour plots for CD28 and CD57 cell surface expression within the CD45RA^−^CCR7^+^CD45RO^+^ central memory compartment. We further investigated senescence-associated loss of CD27 and gain KLRG1, which we observed in conjunction with the above-mentioned senescence indicators (Fig. 4c and d), with a mean 8.1-fold increase in the CD8^+^CD28^lo^CD27^lo^CD57^+^KLRG1^+^ population in MSC co-cultures (Fig. 4d; 4 senescence indicators). Similar trends were observed in indirect co-cultures (Supplementary Fig. 7). Contour plots can be found in Supplementary Figs. 8 and 9. We further assessed the surface expression on MSCs of the CD28 ligands CD80 and CD86, which were negative, as well as HLA-DR which was positive (Supplementary Fig. 10).

## Discussion

As light is shed onto mechanisms of AML-mediated immune escape, a new appreciation has developed regarding the reciprocal interactions within the local tumor microenvironment in suppressing the host immune response, and by extension targeted therapies with antibodies or effector immune cells. MSCs are emblematic of this activity with the ability to express a wide range of soluble and cell surface immune modulating molecules.

Herein, we demonstrated that MSCs significantly abrogate the T-cell-mediated release of several inflammatory molecules and the proliferative potency of AML-targeting T cells, activities which are likely detrimental to sustained, long-term immunotherapeutic response [21–24]. Curiously, even once activated, the paracrine mechanisms of MSCs were found insufficient to prevent directed cytolysis once the immunological synapse has formed, confirming previous findings [7, 25]. As certain MSC-associated molecules such as tumor growth factor *β* reduce both inflammatory and cytolytic molecules at the transcriptional level [26], it could be postulated that MSCs do not affect secretion events per se of granules already present in primed cytotoxic T cells. Alternatively, considering IFNγ promotes cytotoxicity [27], it is possible that reduced inflammation levels still surpass the threshold for efficient T-cell killing activity. As our assays preclude the involvement of direct contact mechanisms such as the PD-1/PD-L1/PD-L2 axis, their role cannot be discounted within the AML microenvironment.

Increase in both exhausted and senescent CD8^+^ lymphocytes has been identified within the peripheral blood and bone marrow of AML patients [16–18]. Exhaustion is denoted by inhibitory receptors such as PD-1, of which we found expression on CAR T cells independently of MSC activity, likely the result of priming and sustained stimulation. With regard to senescence, they can be identified by gain of KLRG1 and CD57, and loss of CD27 and CD28 [28]. We observed loss of CD28 and CD27 and gain of KLRG1 in MSC co-cultures in both CD4^+^ and CD8^+^ fractions. Enrichment of CD8^+^CD28^lo^CD27^lo^CD57^+^KLRG1^+^ cells could also be observed, though they remained at a low percentage relative to the total CD8^+^ population. Curiously, the CD28 ligands CD80 and CD86 are not involved due to lack of expression on MSCs [29], and rather our non-contact cultures imply activity from paracrine mechanisms. It should be noted that senescent T cells in AML have been found to possess increased IFNγ and TNFα potential, though lower IL-2 [18], which differs to what we and many others have demonstrated regarding MSC immunosuppression. It is clear that more study needs to be carried out to robustly conclude if CD28^lo^CD27^lo^CD57^+^KLRG1^+^ T cells under MSC activity are truly senescent. With regard to specific pathways by which MSCs and the AML microenvironment could induce the senescence-associated phenotype, tumor cells have been shown to induce senescence through mediation of cAMP activity within effector T cells [30]. This pathway is potentially shared by MSCs and regulatory T cells (Tregs) via T-cell adenylate cyclase activation by extracellular adenosine produced by the CD73-CD39 receptors [31], as well as through prostaglandin E2 release mediated by COX-2 [32] expressed by both MSCs and myeloid-derived suppressor cells. Indeed, Tregs have been demonstrated to induce CD27 and CD28 loss in both CD4^+^ and CD8^+^ T cells, along with inhibited proliferative capacity [33]. Blocking these pathways could potentially reverse the observed induction of the CD28^lo^CD27^lo^CD57^+^KLRG1^+^ phenotype.

Finally, we demonstrate that MSCs express a plethora of immune modulating factors in response to CAR T-cell activity. We show that IDO-1 inhibition almost completely reverses MSC-mediated suppression of CAR T-cell expansion, though the contribution of other mechanisms should be considered. Indeed, hierarchal clustering of gene expression data from AML bone marrow samples of patients receiving CD3/CD123 bispecific antibody flotetuzumab demonstrated higher immune scores for a number of MSC-associated modulatory signatures such as IDO1, TGFβ, and PDL2, as well a stromal biological signature, in non- and partial responders in comparison with complete responders [34]. Much of the paracrine regulation by MSCs is mediated through extracellular vesicles [35], which, in addition to facilitating the transfer of metabolic and peptidic factors, allow for epigenetic gene regulation via micoRNAs. A large number of extracellular microRNAs have recently been found to be differentially expressed in AML [36], some of which possess potent immunosuppressive activity. This includes bone marrow miR-21 [37], which is also abundantly present in MSC exosomes [38]. In addition to inducing T-cell apoptosis, AML-derived miR-21 can polarize T cells toward the regulatory phenotype very reminiscent of MSC activity [5]. These are just some examples that later could be investigated in relevant preclinical models to develop a combinatorial approach for optimized CAR T-cell therapy.

### Supplementary Information

Below is the link to the electronic supplementary material.

Supplementary file1 (PDF 1836 KB)

## Data Availability

Data are available upon reasonable request.
