# Supplementary material for: Bone marrow-derived mesenchymal stromal cells obstruct AML-targeting CD8+ clonal effector and CAR T-cell function while promoting a senescence-associated phenotype
Source: Cancer Immunol Immunother. 2024 Jan 17;73(1):8. doi: 10.1007/s00262-023-03594-1 (PMC10794426; doi:10.1007/s00262-023-03594-1)
Supplement: Supplementary file 1 — Supplementary file1 (PDF 1836 KB) [file 262_2023_3594_MOESM1_ESM.pdf]

Supplementary Table 1. Flow cytometry antibodies.

Antigen	Fluorophore	Manufacturer	Clone
CCR7	BV421	BD	150503
CD27	BV480	BD	L128
CD28	PE-Cy7	BD	CD28.2
CD28	APC	eBiosciences	CD28.2
CD28	PE-Cy7	BD	CD28.2
CD4	APC	eBioscience	RPA-T4
CD4	V500	BD	RPA-T4
CD4	BUV737	BD	SK3
CD45	V500	BD	HI30
CD45	AF700	BioLegend	HI30
CD45	BUV395	BD	HI30
CD45RA	FITC	eBioscience	HI100
CD45RO	PerCP-eFl710	eBioscience	UCHL1
CD57	PE	BioLegend	QA17A04
CD57	BB515	BD	NK-1
CD80	PerCP-eF710	eBioscience	2D10.4
CD86	BV421	Biolegend	IT2.2
CD8a	APC-eFl780	eBioscience	SK1
CD8a	BUV496	BD	RPA-T8
CD90	PerCP-Cy5.5	eBioscience	eBio5E10
HLA-DR	APC	Miltenyi	REA805
ICAM-1	APC/Fire 750	BioLegend	HA58
KLRG1	RY586	BD	Z7-205.rMAb
LFA-3	BV421	BD	1C3
PD-1	BV421	BioLegend	EH12.2H7
PD-L1	APC	eBioscience	MIH1
PD-L2	PE	eBioscience	MIH18
VCAM-1	PE-Cy7	eBioscience	STA

Supplementary Table 2. Primers.

Gene	Primer Sequence (5’-3’)
GAPDH fwd*	GCAGGGGGGAGCCAAAAGGG
GAPDH rev*	TGCCAGCCCCAGCGTCAAAG
GAPDH fwd	GAAGGTGAAGGTCGGAGTC
GAPDH rev	GAAGATGGTGATGGGATTTC
PTGS2 fwd	TGAGCATCTACGGTTTGCTG
PTGS2 rev	TGCTTGTCTGGAACAACTGC
IDO1 fwd	GCCAGCTTCGAGAAAGAGTTG
IDO1 rev	TGACTTGTGGTCTGTGAGATGA
CD274 fwd	GGCATCCAAGATACAAACTCAA
CD274 rev	CAGAAGTTCCAATGCTGGATTA
PDCD1LG2 fwd	GAGCTGTGGCAAGTCCTCAT
PDCD1LG2 rev	GCAATTCCAGGCTCAACATTA
PDCD1 fwd	CGTGGCCTATCCACTCCTCA
PDCD1 rev	ATCCCTTGTCCCAGCCACTC
ICAM1 fwd	ATGCCCAGACATCTGTGTCC
ICAM1 rev	GGGGTCTCTATGCCCAACAA

* Conventional PCR

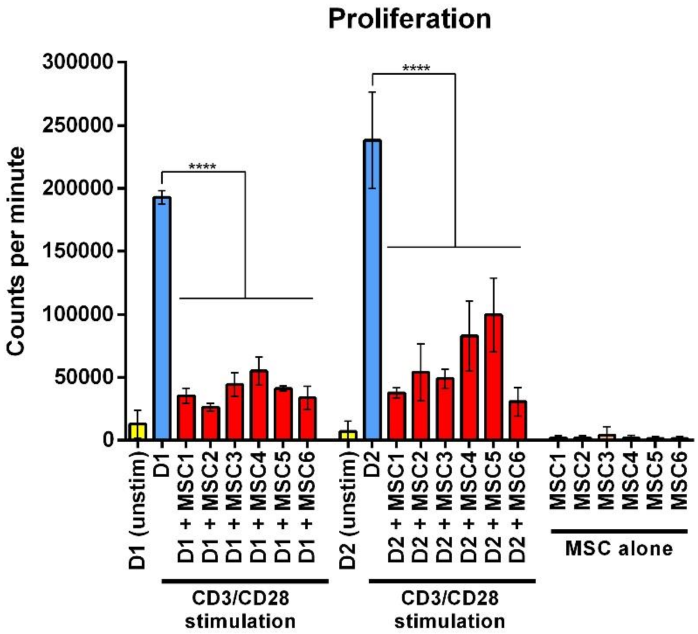

Supplementary Figure 1 MSCs inhibit unmodified T cell proliferation. PBMCs from two healthy donors (D1 and D2) were cultured with or without $5x10^3$ irradiated (30 Gy) allogeneic MSCs from six healthy donors (MSC1-6) at a PBMC:MSC ratio of 100:1 and stimulated with anti-CD3/CD28 antibody-coated beads. Cultures of unstimulated PBMCs and MSC monocultures were also prepared. After 5 days, ^{3}H-radiolabelled thymidine was added and the cultures were incubated for an additional 18h. Cells were harvested and ^{3}H-thymidine incorporation was measured via β-counter, with a readout given in Counts per minute. Data are presented as the mean of technical triplicates ± SD. Asterisks represent statistically significant differences (**** $p \leq 0.0001$)

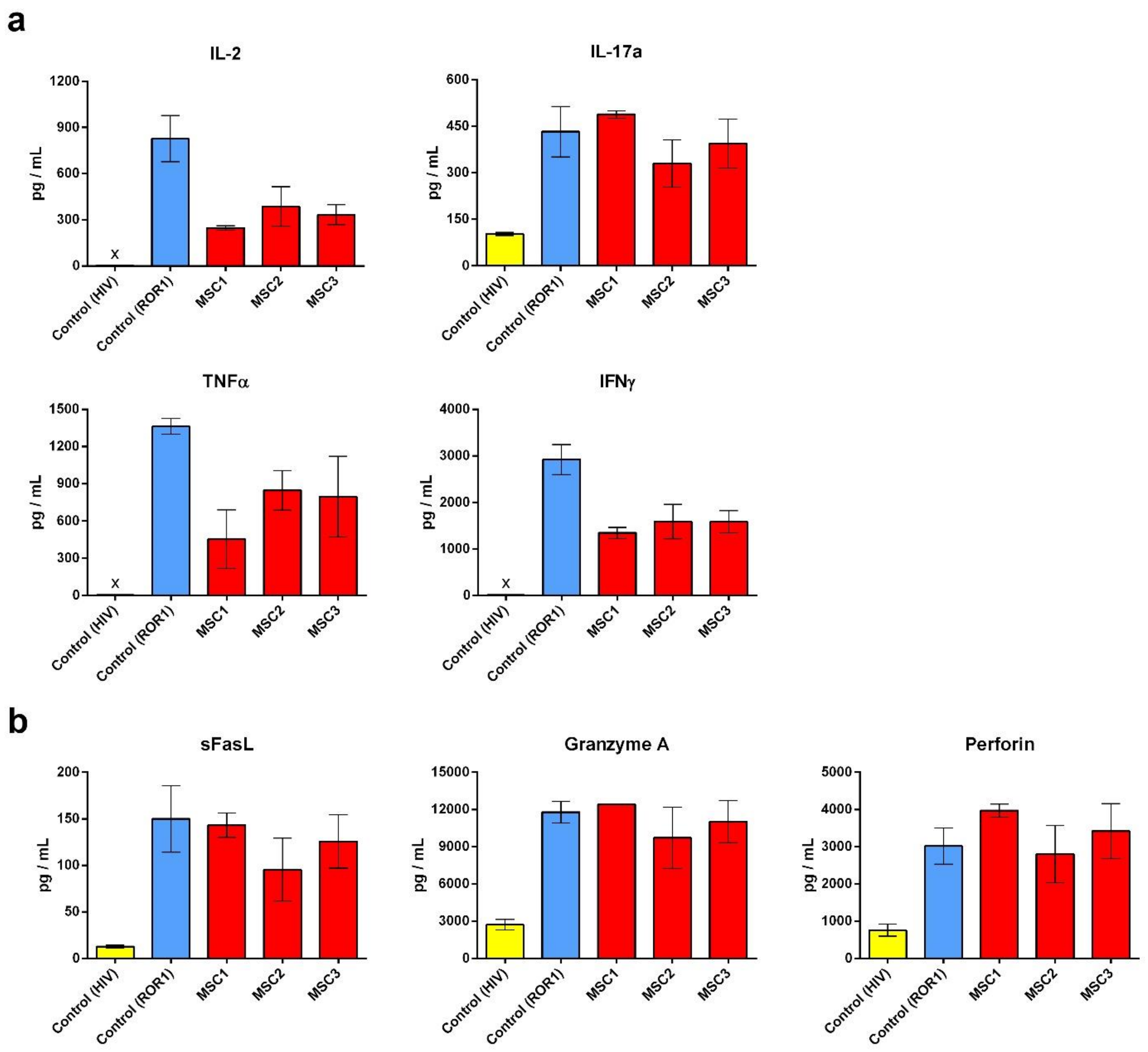

Supplementary Figure 2 MSCs modulate the inflammatory capabilities ROR1-reactive CD8$^+$ T cell clones. Antigen-specific ROR1-reactive CD8$^+$ CTL clones were incubated with target K562 cells pulsed with ROR1 peptide in the presence or absence of 2.5x10^4 allogeneic MSCs from 3 healthy donors (MSC1-3) at a E:T:MSC ratio of 2:10:1. Target cells pulsed with irrelevant HIV Gag-Pol peptides served as a negative control (Control-HIV). After 4h, co-culture supernatant was collected and analyzed for the indicated inflammatory cytokines (a) and cytotoxicity effector molecules (b) via ELISA (presented as mean concentration of technical triplicates ± SD)

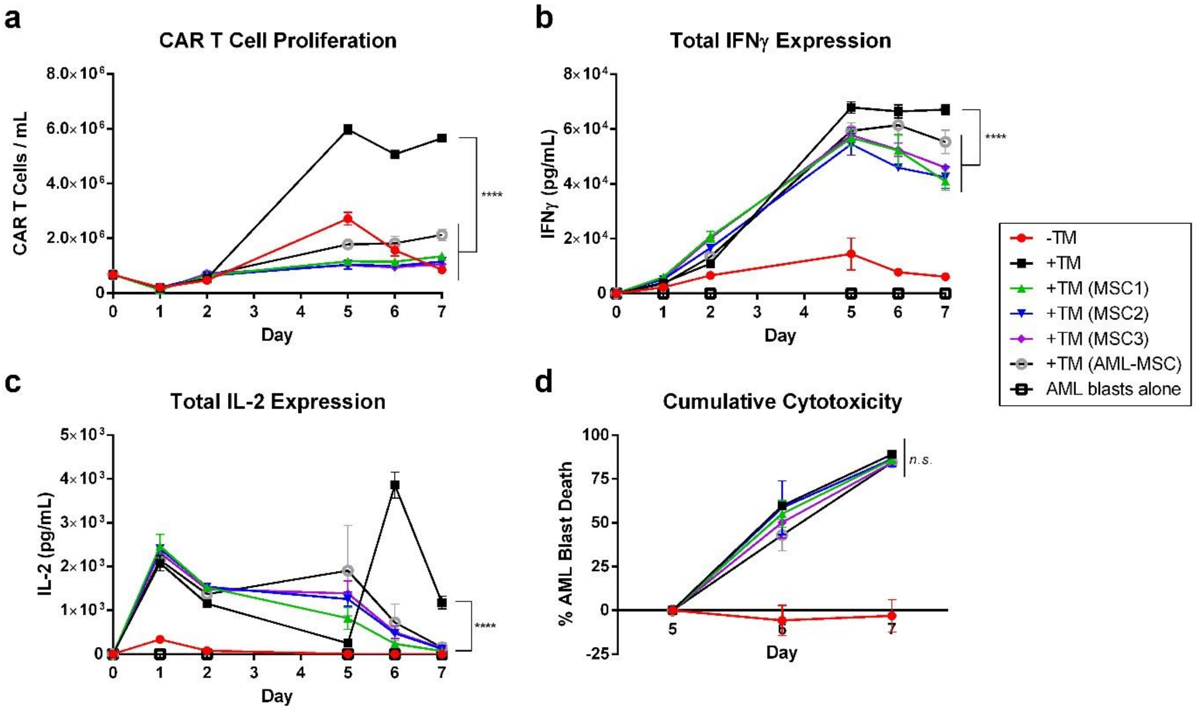

Supplementary Figure 3 MSCs interfere with the proliferative and inflammatory capabilities of CD123-targeting CAR T cells without affecting cytotoxicity. In the apical chamber of the Transwell cell culture system, $eGFP^+$ CAR T cells were redirected with an anti-CD123 target module (+TM, 0.5nM) against eFluor670-labelled $CD123^+$ AML blasts in the presence or absence in the basolateral chamber of $7.5x10^3$ MSCs from three allogeneic healthy donors (MSC1-3) or an allogeneic AML-derived donor (AML-MSC) at a E:T:MSC ratio of 16:16:3. Cultures without TM (-TM) served as a negative control. Additional $eFluor670^+$ blasts were added on day 2 ($1.2x10^5$) and day 5 (variable at an E:T of 1:1.2). Surviving CAR T cells (PI^-eGFP^+) and blasts ($PI^-eFluor670^+$) were quantified via flow cytometry at 1, 2, 5, 6 and 7 days of culture. Data points are presented as the mean of technical triplicates ± SD. Asterisks represent statistically significant differences (**** $p \leq 0.0001$; *n.s.* not significant). **a** CAR T cell proliferation assessed as PI^-eGFP^+ cells/mL over time. **b and c** Supernatant was collected on days 1, 2, 5, 6 and 7 and assessed for IFNγ and IL-2 concentration via ELISA. D) AML blast killing kinetics after the third round of CAR T cell stimulation on day 5 at a E:T of 1:1.2. Data points represent cumulative loss of target cells relative to the initial population on Day 5

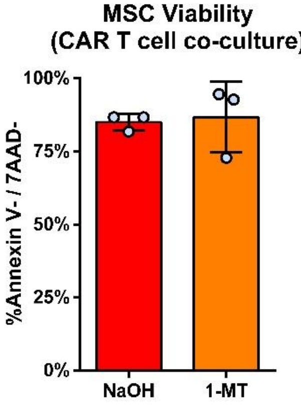

Supplementary Figure 4 MSCs remain viable after 1-MT treatment. In the apical chamber of the Transwell cell culture system, switchable CAR T cells were redirected with an anti-CD123 TM (+TM, 0.5nM) against MOLM-13 cells in the presence or absence in the basolateral chamber of 7.5x10^3 MSCs from three allogeneic healthy donors at a E:T:MSC ratio of 16:16:3. Cultures were treated with 0.2mM NaOH or 0.2mM 1-MT/NaOH. Additional MOLM-13 cells were added on day 2 (6.2x10^4). On day 5, MSCs were recovered and assessed for viability via Annexin V and 7AAD staining, as determined by flow cytometry. Data are presented as the mean of 3 biological replicates ± SD

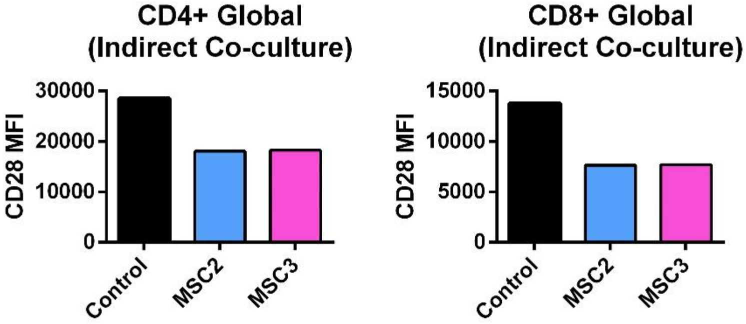

Supplementary Figure 5 MSCs induce senescence of CAR T cells, as characterized by loss of CD28. In the apical chamber of the Transwell cell culture system, eGFP$^+$ switchable CAR T cells were redirected with an anti-CD123 TM (+TM, 0.5nM) against eFluor670-labelled MOLM-13 cells in the presence or absence in the basolateral chamber of 7.5x10^3 MSCs from two allogeneic healthy donors (MSC2 and MSC3) at a E:T:MSC ratio of 16:16:3. Additional eFluor670-labelled MOLM-13 cells were added on day 2 (1.2x10^5) and day 5 (variable at E:T of 1:2). On day 7, surviving CAR T cells (DAPI$^-$eGFP$^+$CD45$^+$) were assessed for CD28 cell surface expression by flow cytometry within the global CD4$^+$ (left) and CD8$^+$ (right) subpopulations. Results are presented as median fluorescence intensity (MFI) of CD28 and are representative of two independent experiments

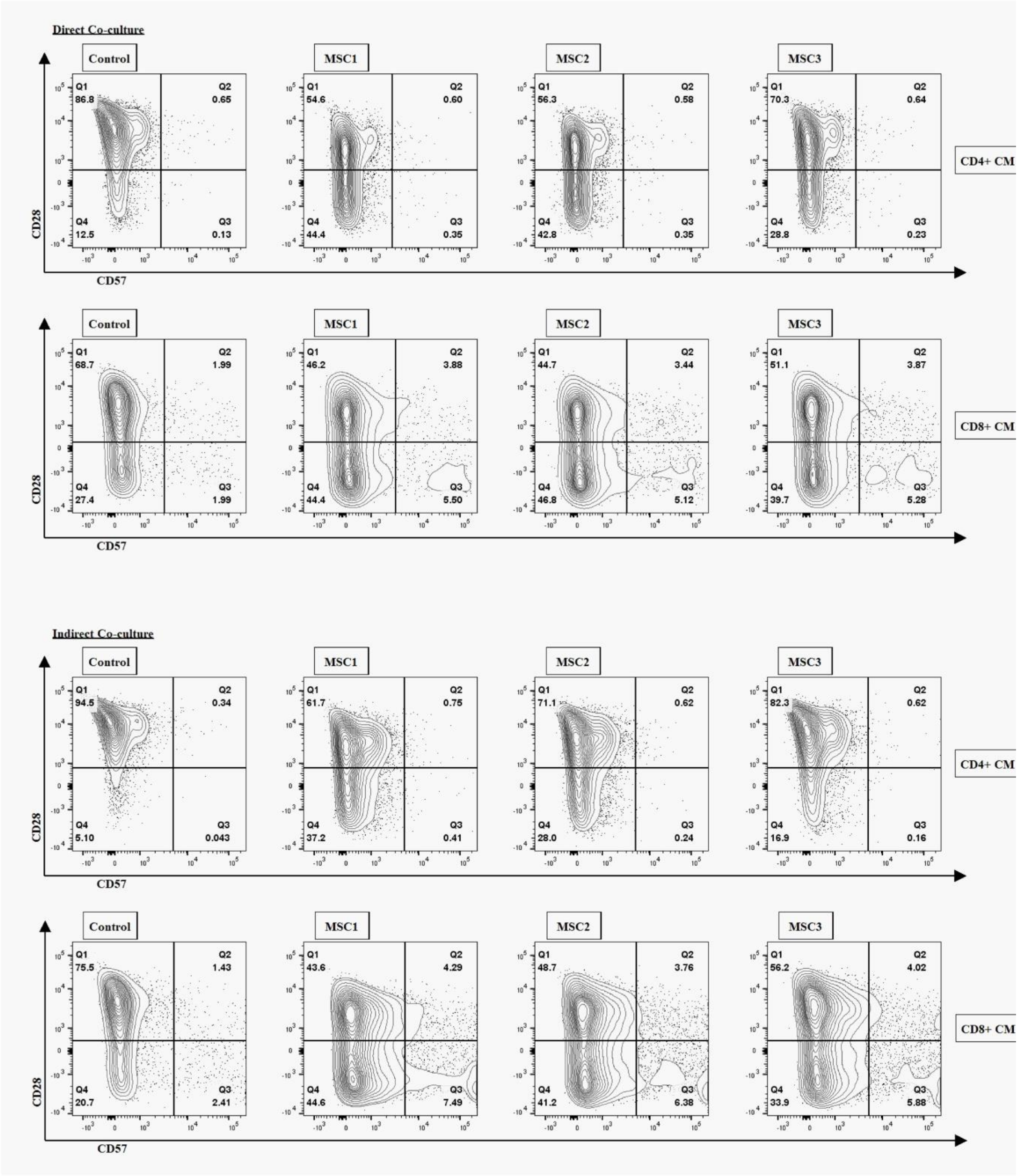

Supplementary Figure 6 MSCs induce senescence of unmodified T cells, as characterized by loss of CD28 and gain of CD57. Example contour plots assessing CD28 and CD57 cell surface expression within the central memory populations ($CD45RA^{-}CCR7^{+}CD45RO^{+}$) of $CD4^{+}$ and $CD8^{+}$ T cells for direct co-cultures (above) and indirect co-cultures (below). Outliers are shown as dots

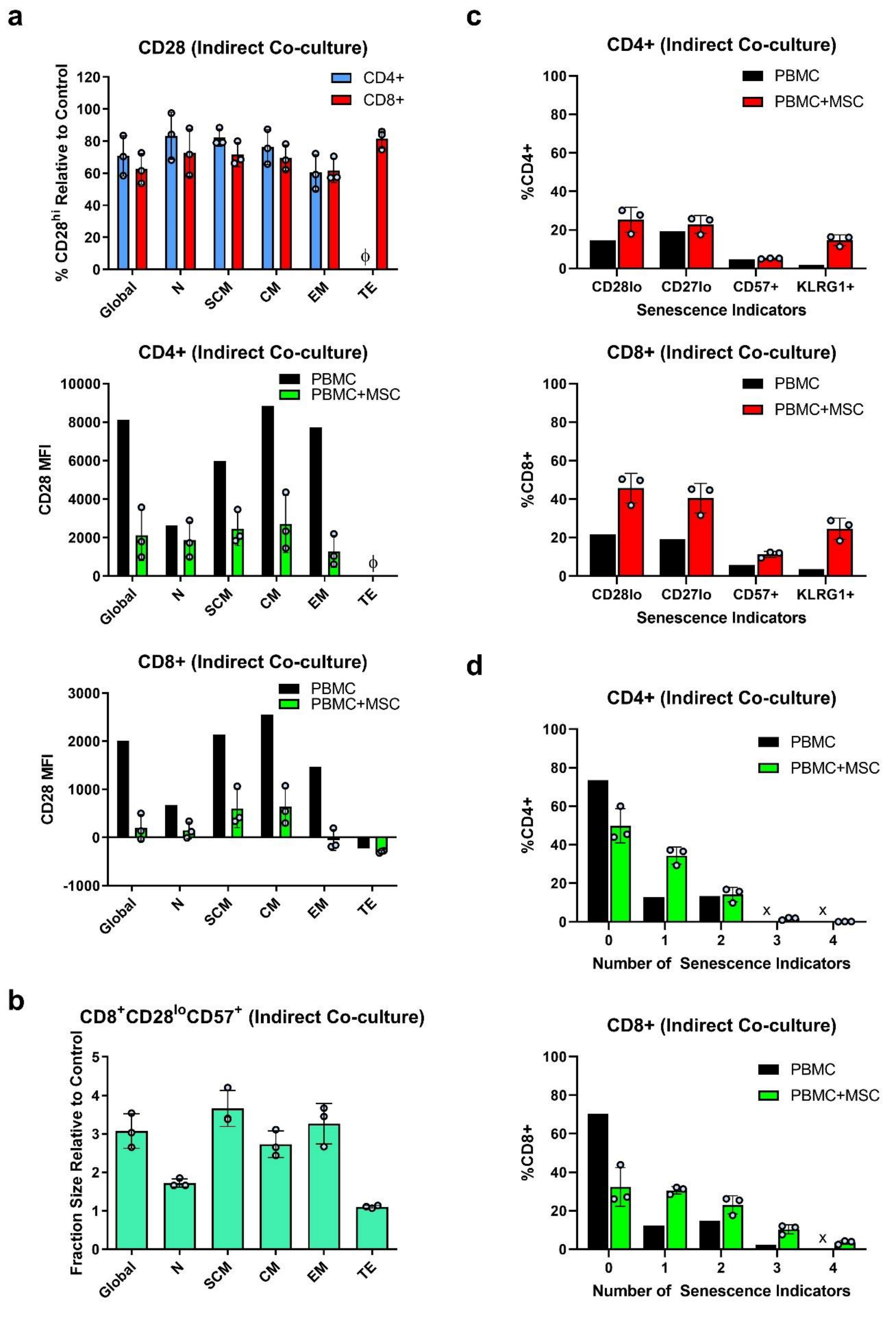

a
CD28 (Indirect Co-culture)
% CD28hi Relative to Control
CD4+
CD8+
Global
N
SCM
CM
EM
TE
ϕ
CD4+ (Indirect Co-culture)
CD28 MFI
PBMC
PBMC+MSC
ϕ
CD8+ (Indirect Co-culture)
CD28 MFI
PBMC
PBMC+MSC
b
CD8+CD28loCD57+ (Indirect Co-culture)
Fraction Size Relative to Control
c
CD4+ (Indirect Co-culture)
%CD4+
PBMC
PBMC+MSC
CD28lo
CD27lo
CD57+
KLRG1+
Senescence Indicators
CD8+ (Indirect Co-culture)
%CD8+
PBMC
PBMC+MSC
Senescence Indicators
d
CD4+ (Indirect Co-culture)
%CD4+
PBMC
PBMC+MSC
x
x
Number of Senescence Indicators
CD8+ (Indirect Co-culture)
%CD8+
PBMC
PBMC+MSC
x
Number of Senescence Indicators

Supplementary Figure 7 MSCs induce senescence of unmodified T cells via paracrine interactions, as characterized by loss of CD28 and CD27, and gain of CD57 and KLRG1. Healthy donor PBMCs (apical chamber) were cultured with or without $5x10^4$ allogeneic MSCs (basolateral chamber) from 3 healthy donors (MSC1-3) at a PBMC:MSC ratio of 5:1 and stimulated with anti-CD3/CD28 antibody-coated beads. After 6 days, cells were harvested and analyzed by flow cytometry. T cell senescence ($CD28^{lo}$, $CD27^{lo}$, $CD57^{+}$, $KLRG1^{+}$) was assessed within the global $CD4^{+}$ and $CD8^{+}$ T cell populations (Global; $DAPI^{-}CD45^{+}CD4^{+}$ and $DAPI^{-}CD45^{+}CD8^{+}$), as well as within further T cell memory subpopulations: Naïve (N; $CD45RA^{+}CCR7^{+}CD45RO^{-}$), Stem Cell Memory (SCM; $CD45RA^{+}CCR7^{+}CD45RO^{+}$), Central Memory (CM; $CD45RA^{-}CCR7^{+}CD45RO^{+}$), Effector Memory (EM; $CD45RA^{-}CCR7^{-}CD45RO^{+}$) and Terminal Effector (TE; $CD45RA^{+}CCR7^{-}CD45RO^{-}$). **a** Above: Fraction size of $CD28^{hi}$ cells within the global and memory stages of $CD4^{+}$ (blue) and $CD8^{+}$ (red) T cell population in direct MSC co-cultures relative to the control. Below: Median fluorescence intensity (MFI) of CD28 within the global and memory stages of $CD4^{+}$ (above) and $CD8^{+}$ (below) T cell populations for control (black) MSC co-cultures (green). Data are presented as the mean of biological triplicates ± SD. φ indicates lack of sufficient number of events for assessment. **b** Fraction size of $CD28^{lo}CD57^{+}$ cells within the global and memory stages of the $CD8^{+}$ T cell population in direct MSC co-cultures relative to the control. Results are representative of three independent experiments. **c** Fraction size of each senescence marker within the global $CD4^{+}$ (above) and $CD8^{+}$ (below) populations for control (black) and MSC co-cultures (red). **d** Fraction size based on the number of co-expressed senescence indicators within the global $CD4^{+}$ (above) and $CD8^{+}$ (below) populations for control (black) and MSC co-cultures (green). Data are presented as the mean of biological triplicates ± SD

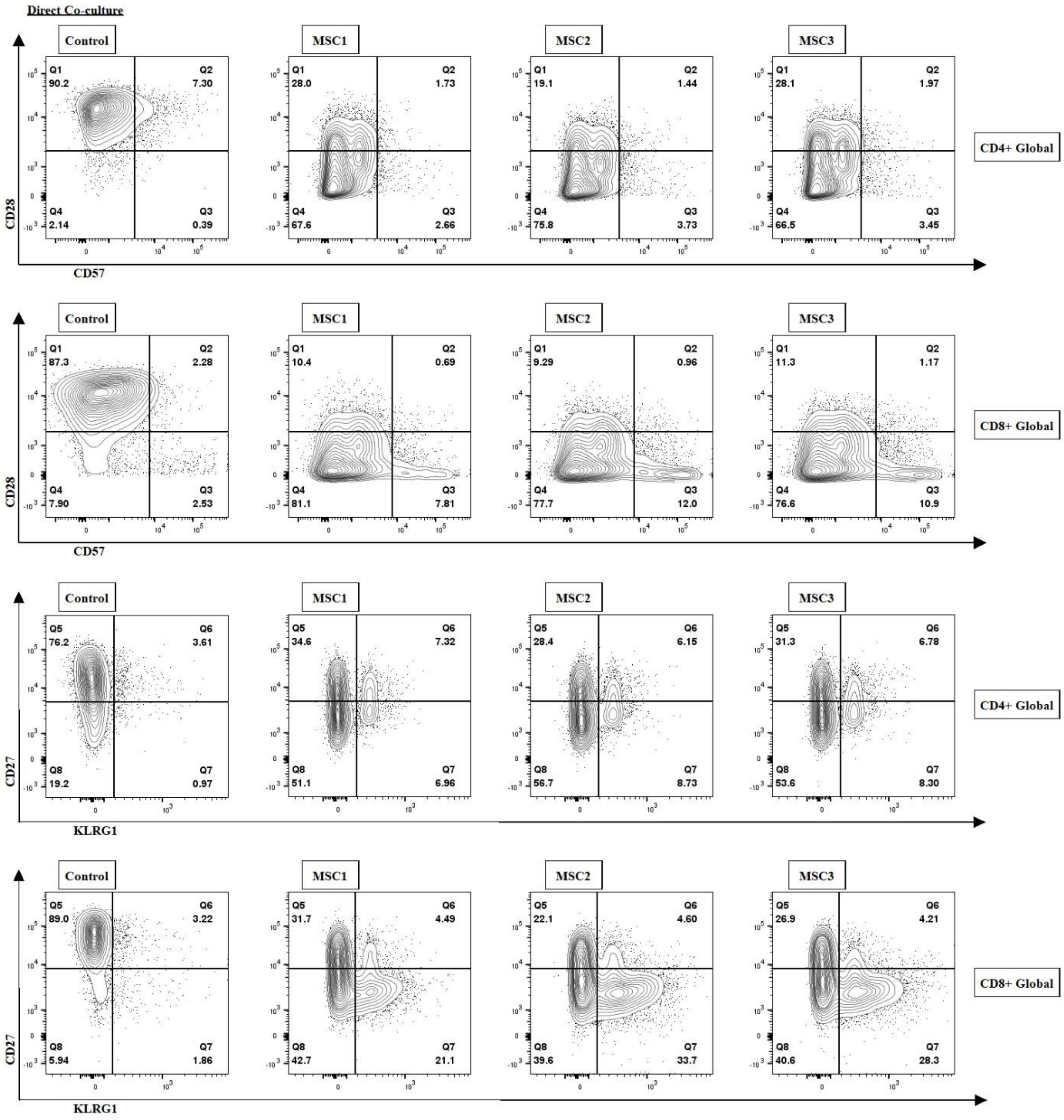

Supplementary Figure 8 MSCs induce senescence of unmodified T cells, as characterized by loss of CD28 and CD27, and gain of CD57 and KLRG1. Contour plots assessing CD28, CD57, CD27 and KLRG1 cell surface expression within CD4^{+} and CD8^{+} T cell populations in direct co-cultures with MSCs

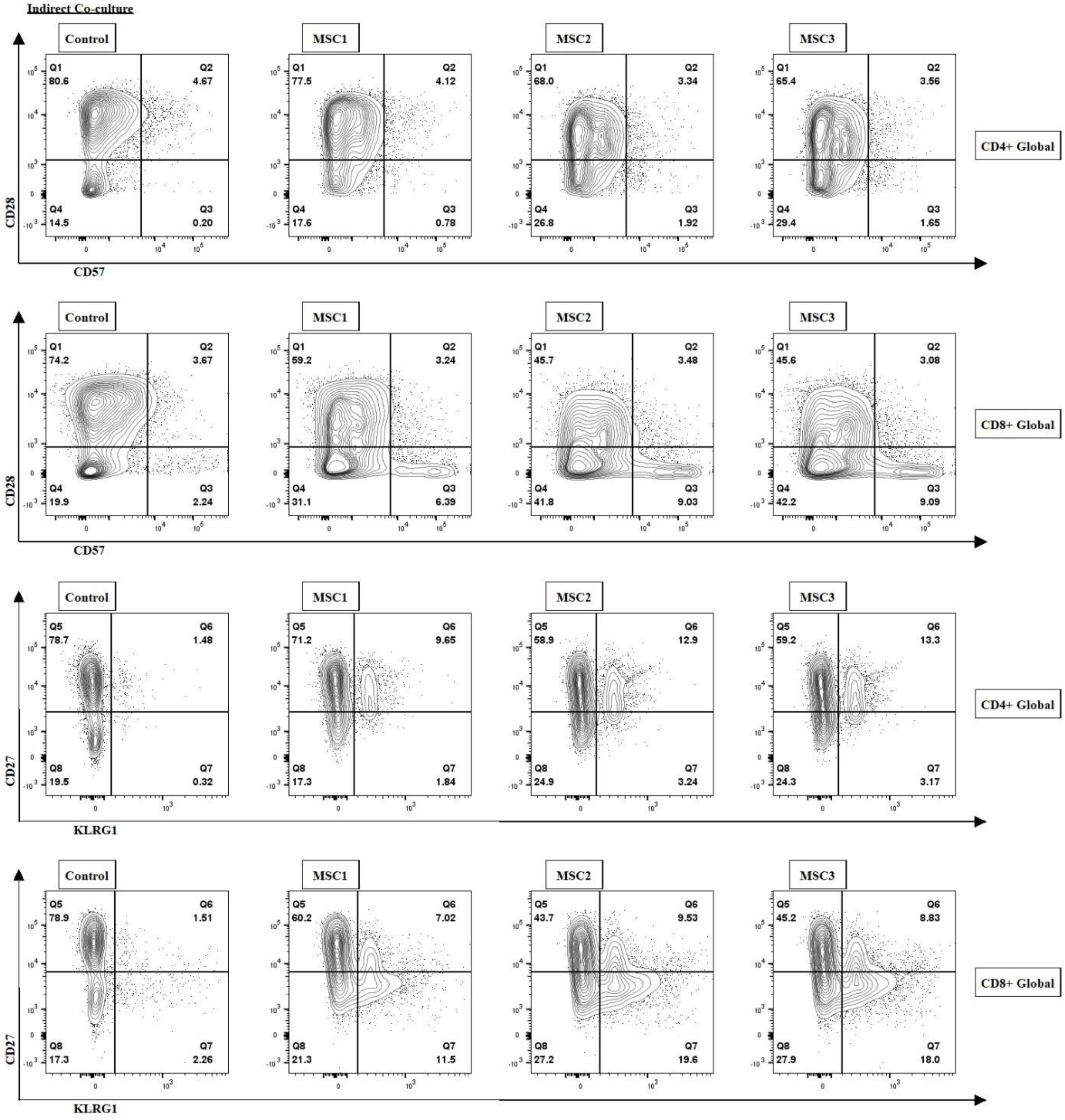

Supplementary Figure 9 MSCs induce senescence of unmodified T cells via paracrine interactions, as characterized by loss of CD28 and CD27, and gain of CD57 and KLRG1. Contour plots assessing CD28, CD57, CD27 and KLRG1 cell surface expression within $CD4^+$ and $CD8^+$ T cell populations in indirect co-cultures with MSCs

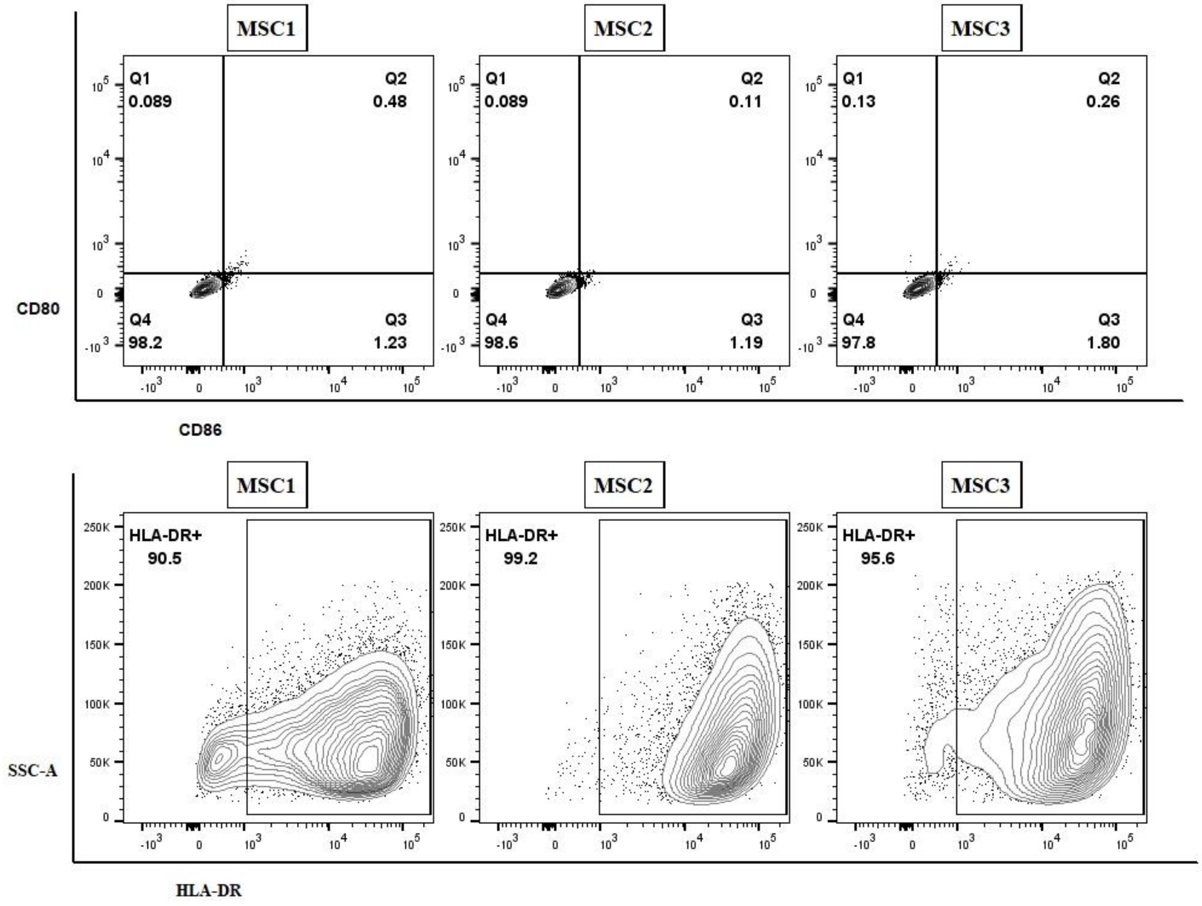

Supplementary Figure 10 MSCs can express HLA-DR, but not the CD28 ligands CD80 and CD86, in response to activated PBMCs. In the apical chamber of the Transwell cell culture system, CD3/CD28-stimulated healthy donor PBMCs were cultured in the presence in the basolateral chamber of $5x10^4$ allogeneic MSCs from 3 healthy donors (MSC1-3) at a PBMC:MSC ratio of 5:1. After 6 days, the MSCs were recovered and assessed for cell surface expression of CD80, CD86 and HLA-DR by flow cytometry. Data are presented as contour plots with outliers as dots

List of Abbreviations

1-MT	1-methyl-L-tryptophan
AML	Acute myeloid leukemia
CAR	Chimeric antigen receptor
COX-2	Cyclooxygenase 2
CTL	Cytotoxic T cell
DAPI	4′,6-diamidino-2-phenylindole
E:T	Effect-to-target ratio
ELISA	Enzyme-linked immunosorbent assay
FACS	Fluorescence-associated cell sorting
FBS	Fetal bovine serum
GAPDH	Glyceraldehyde-3-phosphate dehydrogenase
ICAM-1	Intercellular adhesion molecule 1
IDO1	Indoleamine 2,3-dioxygenase 1
IFNγ	Interferon gamma
IL-2	Interleukin 2
KLRG-1	Killer cell lectin-like receptor G1
LFA-3	Lymphocyte function-associated antigen 3
MSC	Mesenchymal stromal cell
PBMC	Peripheral blood mononuclear cells
PD-1	Programmed cell death 1
PDCD1LG2	Programmed cell death 1 ligand 2
PD-L	Programmed death-ligand
PGE2	Prostaglandin E2
PI	Propidium iodide
PTGS2	Prostaglandin-endoperoxide synthase 2
ROR1	Tyrosine-protein kinase transmembrane receptor 1
sFasL	Soluble Fas ligand
TM	Target module
TGFβ	Tumour growth factor beta
TNFα	Tumour necrosis factor alpha
VCAM-1	Vascular cell adhesion protein 1
WT1	Wilm’s tumour protein 1
